# Surgery for Intraductal Papillary Mucinous Neoplasms of the Pancreas: Preoperative Factors Tipping the Scale of Decision-Making

**DOI:** 10.1245/s10434-022-11326-5

**Published:** 2022-01-24

**Authors:** Giovanni Marchegiani, Stefano Crippa, Giampaolo Perri, Paola M. V. Rancoita, Andrea Caravati, Giulio Belfiori, Tommaso Dall’Olio, Francesca Aleotti, Stefano Partelli, Claudio Bassi, Massimo Falconi, Roberto Salvia

**Affiliations:** 1 Department of General and Pancreatic Surgery, Verona University Hospital, Università degli Studi di Verona, Verona, Italy; 2 Division of Pancreatic Surgery, Pancreas Translational and Clinical Research Center, IRCCS San Raffaele Scientific Institute, Università Vita-Salute, Milan, Italy; 3 University Centre of Statistics in the Biomedical Sciences, Vita-Salute San Raffaele University, Milan, Italy

## Abstract

**Background:**

Decision-making in intraductal papillary mucinous neoplasms (IPMNs) of the pancreas depends on scaling the risk of malignancy with the surgical burden of a pancreatectomy. This study aimed to develop a preoperative, disease-specific tool to predict surgical morbidity for IPMNs.

**Methods:**

Based on preoperative variables of resected IPMNs at two high-volume institutions, classification tree analysis was applied to derive a predictive model identifying the risk factors for major morbidity (Clavien–Dindo ≥3) and postoperative pancreatic insufficiency.

**Results:**

Among 524 patients, 289 (55.2%) underwent pancreaticoduodenectomy (PD), 144 (27.5%) underwent distal pancreatectomy (DP), and 91 (17.4%) underwent total pancreatectomy (TP) for main-duct (18.7%), branch-duct (12.6%), or mixed-type (68.7%) IPMN. For 98 (18.7%) of the patients, major morbidity developed. The classification tree distinguished different probabilities of major complications based on the type of surgery (area under the surve [AUC] 0.70; 95% confidence interval [CI], 0.63–0.77). Among the DP patients, the presence of preoperative diabetes identified two risk classes with respective probabilities of 5% and 25% for the development of major morbidity, whereas among the PD/TP patients, three different classes with respective probabilities of 15%, 20%, and 36% were identified according to age and body mass index (BMI). Overall, history of diabetes, age, and cyst size segregated three different risk classes for new-onset/worsening diabetes.

**Conclusions:**

In presumed IPMNs, the disease-specific risk of major morbidity and pancreatic insufficiency can be determined in the preoperative setting and used to personalize the possible surgical indication. Age and overweight status in case of PD/TP and diabetes in case of DP tip the scale toward less aggressive clinical management in the absence of features suggestive for malignancy.

**Supplementary Information:**

The online version contains supplementary material available at 10.1245/s10434-022-11326-5.

Due to growing knowledge on the biologic behavior of intraductal papillary mucinous neoplasms (IPMNs) of the pancreas, indications for surgery are increasingly reserved for selected cases.^1^ Whereas the indication for surgical resection in cases of main duct involvement is more consolidated, the treatment for branch-duct IPMNs (BD-IPMNs) remains controversial and varies by morphology. As a matter of fact, approximately only 2% of patients with presumed BD-IPMNs are referred to surgery at high-volume centers after clinical evaluation.

The international consensus guidelines subcategorize BD-IPMNs into those with low risk, those with “worrisome features,” and those with high-risk stigmata, with an increasing associated likelihood of malignancy. Early surgery is advised only for the latter category.^2^ This cautious approach aims to select only candidates with appropriate oncologic targets (high-grade dysplasia or invasive carcinoma), sparing the burden of unnecessary surgery for patients who do not show clinical or radiographic signs of malignancy. Pancreatic surgery is indeed still burdened by a high rate of major morbidity, ranging from 24 to 30%, and a high rate of major mortality, ranging from 1 to 5%.^3–6^

Notably, most data regarding surgical morbidity and mortality after pancreatectomy are derived from surgical series including different types of pathologies, with IPMNs accounting for only a minor proportion of the patients. The larger part of major morbidity in pancreatic surgery is driven by pancreas-specific complications, particularly postoperative pancreatic fistula (POPF).^7,8^

Several prediction models have been developed in an attempt to identify risk factors for POPF, with the fistula risk score (FRS) being the most widely accepted and validated.^9^ However, this score relies on intraoperative parameters such as main pancreatic duct (MPD) diameter, gland texture, and estimated blood loss (EBL), substantially limiting its value in the preoperative setting. Despite the potential implications for clinical management, together with disease morphologic features and patient life expectancy, the assessment of the specific surgical risk is not currently available in the preoperative setting for patients affected by IPMNs.

This study aimed to define the surgical outcomes of pancreatectomy for IPMN of the pancreas, to identify independent predictors for major postoperative morbidity, and to develop a preoperative disease-specific tool to predict the likelihood of major morbidity and postoperative pancreatic insufficiency.

## Methods

### Inclusion Criteria

This bicentric retrospective study was approved by the institutional review board (approval no. 1101CESC, Comitato Etico delle Province di Verona e Rovigo, informed consent waived). All patients undergoing surgery for pathologically proven IPMN at the Department of General and Pancreatic Surgery–The Pancreas Institute, University of Verona Hospital Trust, and at Pancreatic Surgery, San Raffaele Scientific Institute, Milan, from 2012 to 2018 were screened for eligibility.

Pathologic evaluations, performed by specialized pancreatic pathologists at the two participant institutions, are reported according to the standardized nomenclature of pancreatic pathology on IPMN.^10,11^ Data were retrieved from prospectively maintained institutional databases and included patient characteristics, clinical and radiologic features, postoperative course, pathologic diagnosis, and follow-up data. Patients who underwent atypical pancreatic resections (enucleation or middle pancreatectomy) and patients with missing data in the main outcome of interest (Clavien–Dindo) were excluded.

### Definition of Preoperative Variables

The included patients underwent at least preoperative cross-sectional imaging (either computed tomography [CT] scan or magnetic resonance imaging [MRI]), allowing us to define the preoperative features of the cyst. Definitions of worrisome features (WFs) and high-risk stigmata (HRS) were derived from the International Association of Pancreatology guidelines.^2^ Cystic features were recorded according to the type of radiologic imaging. When multiple imaging studies were performed, the largest size was considered, and the maximum diameter of the cyst was reported. Information regarding the comorbidities of the enrolled patients was retrospectively retrieved from the patient charts using manual reviews of the clinical history. The Charlson Age Comorbidity Index (CACI)^12^ was calculated by adding the comorbidity score and the age-adjusted comorbidity index. The comorbidity score was calculated at the time of pancreatic cancer diagnosis based on the clinical history.

### Definition of Postoperative Outcomes

Postoperative morbidity was defined according to the Clavien–Dindo (CD) classification, and severe postoperative morbidity was defined as a CD score of 3 or higher.^13^ Postpancreatectomy hemorrhage (PPH), POPF, and delayed gastric emptying (DGE) were defined according to the International Study Group on Pancreatic Surgery (ISGPS) definitions.^14–16^ Clinically relevant POPF (CR-POPF) was defined as grade B or C POPF. Bile leak (BL) was defined according to the International Study Group on Liver Surgery (ISGLS).^17^ Sepsis and septic shock definitions were consistent with the Sepsis-3 consensus definition.^18^ Mortality was defined at 90 days.

The definition of postoperative new onset of diabetes was based on the reporting of a normal preoperative free-blood glucose (FBG) or HbA1c, and postoperatively by measured glucose metabolism including FBG/HbA1c level and/or insulin medication. The postoperative worsening of a pre-existent diabetes was defined as a change in the therapeutic management of diabetes from the preoperative status. Pancreatic exocrine insufficiency was defined according to the presence of steatorrhea and the necessity of enzyme treatment, with cessation/mitigation of diarrhea after enzyme supplementation.

### Statistical Analysis

Continuous variables were summarized with medians and interquartile ranges (IQRs), whereas categorical variables were summarized as absolute and relative frequencies. Time to new-onset diabetes (NOD) or worsening diabetes was described with the Kaplan–Meier curve. Comparisons between categorical variables were performed with Fisher’s exact test.

Univariate regression analyses were performed to evaluate the separate roles of demographic and clinical patient characteristics in predicting surgical short- and long-term complications. Logistic regression was used to predict a Clavien–Dindo score of 3 or higher or exocrine insufficiency within 1 month after pancreatic resection, whereas Cox proportional hazards regression was used to predict the time to NOD or worsening diabetes. To account for multiple testing, the *P* values in the univariate analysis were adjusted with Bonferroni’s correction.

Decision trees were estimated to derive a predictive model to identify patients with a different risk of outcome occurrence using the same demographic and clinical patient characteristics as in the univariate regression analysis. Depending on the type of outcome, the classification tree (for binary outcomes; i.e., Clavien–Dindo ≥3 vs <3 or the occurrence of exocrine insufficiency within 1 month after pancreatic resection) or the survival tree (for the time to NOD or worsening diabetes) was applied. In general, the decision tree internally selects the best cut points for the continuous variables used in the model, as well as which variables must be retained in the predictive model.

In all the analyses, the algorithm was set to derive risk groups of at least 20 patients. Finally, for each of the identified risk groups of patients, a probability of occurrence of the event was computed in case of binary outcomes, or descriptive statistics of the survival curves were derived for the time to NOD or worsening diabetes. In the case of binary outcomes, the goodness of the predictive model was evaluated by deriving the receiver operating characteristic (ROC) curve of the predicted probabilities obtained from the model and by computing the corresponding area under the curve (AUC). In the case of the time-to-event outcome, the goodness of the model was assessed by computing the C-index of Cox’s proportional model with a covariate representing the discovered risk groups. The confidence interval of the C-index was computed using 500 bootstrap resamples. In the analysis of the time to NOD or worsening diabetes, all TPs were excluded, whereas in the analysis of the occurrence of exocrine insufficiency within 1 month after pancreatic resection, all patients who underwent TP or had chronic pancreatitis were excluded.

Missing data were not imputed. Thus, each analysis considered only complete cases for the variables used in the analysis except for the decision tree analyses, which could handle missing data. All *P* values lower than 0.05 were considered significant. All confidence intervals (CIs) were computed at the 95% confidence level. All statistical analyses were performed using R3.5.0 (http://www.rproject.com), and decision trees were estimated with the ‘‘rpart’’ R package (The R Foundation, Vienna, Austria).

## Results

The study included 524 patients. Eight patients were excluded because they underwent enucleation (*n* = 3) or middle pancreatectomy (*n* = 2) or because of missing data in the primary outcome (*n* = 3). Pancreaticoduodenectomy (PD) was performed for 289 patients (55.2%), total pancreatectomy (TP) for 91 patients (17.4%), and distal pancreatectomy (DP) for 144 patients (27.5%).

### Clinical Characteristics of the Study Population

The demographics and clinical radiologic characteristics of the sample are shown in Table 1. The median age was 68 years (IQR, 14 years), and the median body mass index (BMI) was 24.2 kg/m (IQR, 4.29 kg/m^2^). Preoperatively, 96 patients (18.5%) had diabetes, whereas 130 patients (28.3%) had American Society of Anesthesiology (ASA) scores of 3 or higher. The median CACI was 3 (IQR, 2).

**Table 1:** Sample features: distribution is overall and by final pathology (*n* = 524)

	*n* (%)	LGD	HGD/inv	*P* value
		*n* (%)	*n* (%)	
Sex (male)	276 (52.7)	89 (51.4)	187 (53.3)	0.693
Median age: years (IQR)	68 (14)	68 (15)	69 (14)	0.198
Median BMI: kg/m^2^ (IQR)	24.2 (4.29)^a^	23.9 (4.5)	24.2 (4.3)	0.792
Current smoker	92 (18)^b^	36 (20.8)	56 (16.6)	0.173
Comorbidities				
Diabetes	96 (18.5)^c^	26 (15.1)	70 (20.2)	0.163
Hypertension	276 (52.7)	87 (50.3)	189 (53.8)	0.443
Solid tumor	68 (13)	24 (13.9)	44 (12.5)	0.668
Median CACI (IQR)	3 (2)	3 (2)	3 (2)	0.167
ASA ≥3	130 (28.3)^d^	28 (19.7)	102 (32.1)	0.007
Symptoms				
Jaundice	80 (15.3)^e^	6 (3.5)	74 (21.2)	<0.001
Abdominal pain	163 (31.2)^f^	55 (31.8)	108 (30.9)	0.828
Weight loss	132 (25.2)^f^	32 (18.5)	100 (28.6)	0.013
Acute pancreatitis	102 (19.5)^f^	44 (25.4)	58 (16.6)	0.016
Median cyst size: mm (IQR)	30 (20)^g^	30 (15)	30 (24)	0.060
MPD dilation ≥5 mm	385 (84.2)^h^	120 (79.5)	265 (86.6)	0.069
Mural nodules**	114 (22.1)^i^	42 (24.6)	72 (20.9)	0.341
Solid component	227 (43.7)^j^	27 (15.8)	200 (57.3)	<0.001
Chronic pancreatitis	26 (5.0)^k^	8 (4.7)	18 (5.1)	0.819
WFs	476 (91.0)^f^	158 (91.9)	318 (90.6)	0.635
HRSs	327 (62.5)^f^	71 (41.3)	256 (72.9)	<0.001

*LGD* low-grade dysplasia, *HGD/inv* high-grade dysplasia/invasive cancer, *IQR* interquartile range, *BMI* body mass index, *CACI* Charlson-Age Comorbidity Index, *ASA* American Society of Anesthesiologists, *MPD* main pancreatic duct, *WFs* worrisome features, *HRSs* high-risk stigmata

Missing data: ^a^129; ^b^13; ^c^5; ^d^64; ^e^2; ^f^1; ^g^142; ^h^67; ^i^8; ^j^4; ^k^3

The most common symptom was abdominal pain (31.2%), followed by weight loss (25.2%) and acute pancreatitis (19.5%). The median cyst size was 30 mm (IQR, 20 mm). Main pancreatic duct (MPD) dilation was observed in 84.2% of the patients. Overall, 91% of the patients presented with at least one worrisome feature (WF), and 62.5% had at least one high-risk stigmata (HRS). A solid component, jaundice, weight loss, and the presence of HRS were significantly associated with the presence of high-grade dysplasia/invasive cancer. The pathologic diagnoses are reported in Table 2.

**Table 2:** Pathologic features (*n* = 524)

	*n* (%)
Final pathology	
MD-IPMN	98 (18.7)
MT-IPMN	360 (68.7)
BD-IPMN	66 (12.6)
Invasive IPMN	211 (40.3)
Non-invasive IPMN	313 (59.7)
LGD	173 (55.3)
HGD	140 (44.7)

*IPMN* intraductal papillary mucinous neoplasm, *MD-IPMN* main-duct IPMN, *MT-IPMN* mixed-type IPMN, *BD-IPMN* branch-duct IPMN, *LGD* low-grade dysplasia, *HGD* high-grade dysplasia

### Postoperative Outcomes

The postoperative outcomes are summarized in Table 3. Overall, postoperative morbidity occurred for nearly half of the patients (44.8%), without a significant difference among the types of surgery (*P* = 0.0564). Overall, CR-POPF occurred for 52 patients (14.6%) and was more frequent after PD than after DP (15.2% vs 5.5%; *P* = 0.0026). Notably, no grade C POPF occurred among the patients who underwent DP. In only one patient (0.7%) was DGE anecdotal after DP, compared with 8.0% of the patients who underwent PD and 11.1% of the patients who underwent TP (*P* = 0.0004). Overall, 98 patients (18.7%) had a CD of 3 or higher.

**Table 3:** Postoperative outcomes: distribution is overall, by surgical treatment group, and by final pathology (*n* = 524)

	Total (*n* = 524)	PD (*n* = 289) (55.2%)	DP (*n* = 144) (27.5%)	TP (*n* = 91) (17.4%)	LGD (*n* = 173) (33.1%)	HGD/inv (*n* = 351) (66.9%)	*P* value
	*n* (%)	*n* (%)	*n* (%)	*n* (%)	*n* (%)	*n* (%)	
Overall morbidity	235 (44.8)	143 (49.5)	58 (40.3)	34 (37.4)	77 (44.5)	158 (45.1)	0.912
Surgical morbidity							
Biochemical leak	76 (21.3)	26 (9.0)	49 (34.0)	1 (1.1)	35 (20.2)	41 (11.7)	0.009
CR-POPF	52 (14.6)	44 (15.2)	8 (5.5)	0 (0)	21 (12.1)	31 (8.8)	0.234
PPH	57 (10.9)	35 (12.1)	10 (6.9)	12 (13.2)	22 (12.7)	35 (10.0)	0.348
DGE	34 (6.5)^a^	23 (8.0)	1 (0.7)	10 (11.1)^a^	11 (6.4)	23 (6.6)	0.939
Abdominal abscess	19 (3.6)	14 (4.8)	3 (2.1)	2 (2.2)	8 (4.6)	11 (3.1)	0.394
Biliary fistula	37 (7.1)	27 (9.3)	3 (2.1)	7 (7.7)	10 (5.8)	27 (7.7)	0.417
Enteric fistula	21 (4.0)	15 (5.2)	3 (2.1)	3 (3.3)	6 (3.5)	15 (4.3)	0.654
Chyle leak	19 (3.6)	10 (3.5)	3 (2.1)	6 (6.6)	4 (2.3)	15 (4.3)	0.259
Medical morbidity							
Sepsis	27 (5.2)	19 (6.6)	4 (2.8)	4 (4.4)	4 (2.3)	23 (6.6)	0.038
Respiratory complications	71 (13.5)	42 (14.5)	18 (12.5)	11 (12.1)	26 (15.0)	45 (12.8)	0.487
Cardiac complications	24 (4.6)	13 (4.5)	6 (4.2)	5 (5.5)	9 (5.2)	15 (4.3)	0.632
Acute renal failure	11 (2.1)	7 (2.4)	2 (1.4)	2 (2.2)	4 (2.3)	7 (2.0)	0.815
Neurologic complications	1 (0.2)	1 (0.3)	0 (0)	0 (0)	1 (0.6)	0 (0)	0.154
Surgical-site infections	15 (2.9)	9 (3.1)	2 (1.4)	4 (4.4)	2 (1.2)	13 (3.7)	0.099
Clavien–Dindo score							
No	112 (21.4)	60 (20.8)	28 (19.4)	24 (26.4)	31 (17.9)	81 (23.1)	0.176
1	153 (29.2)	80 (27.7)	52 (36.1)	21 (23.1)	57 (32.9)	96 (27.4)	0.185
2	161 (30.7)	81 (28.0)	52 (36.1)	28 (30.8)	56 (32.4)	105 (30.0)	0.567
3a	49 (9.4)	34 (11.8)	6 (4.2)	9 (9.9)	17 (9.8)	32 (9.1)	0.792
3b	23 (4.4)	13 (4.5)	4 (2.8)	6 (6.6)	6 (3.5)	17 (4.8)	0.469
4a	8 (1.5)	5 (1.7)	2 (1.4)	1 (1.1)	2 (1.2)	6 (1.7)	0.627
4b	4 (0.8)	3 (1.0)	0 (0)	1 (1.1)	1 (0.6)	3 (0.9)	0.732
5	14 (2.7)	13 (4.5)	0 (0)	1 (1.1)	4 (2.3)	10 (2.9)	0.720
Clavien–Dindo ≥3	98 (18.7%)	68 (23.5)	12 (8.3)	18 (19.8)	30 (17.3)	68 (19.4)	0.574
Median LOS: days (IQR)	10 (6)^b^	10 (7)^c^	8 (4)^d^	11 (9)^d^	9 (7)	10 (6)	0.010
Endocrine insufficiency							
New onset	111 (25.6)^e^	68 (23.5)	43 (29.9)	–	41 (23.7)	70 (19.9)	0.322
Worsening	64 (66.7)^e,f^	34 (65.4)^f^	17 (70.8)^f^	–	18 (10.4)	46 (13.1)	0.375
Exocrine insufficiency	156 (36.0)^g^	120 (41.5)	36 (25.0)	–	56 (32.4)	100 (28.5)	0.361

*PD* pancreaticoduodenectomy, *DP* distal pancreatectomy, *TP* total pancreatectomy, *LGD* low-grade dysplasia, *HGD/inv* high-grade dysplasia/invasive cancer, *CR-POPF* clinically relevant postoperative pancreatic fistula, *PPH* post-pancreatectomy hemorrhage, *DGE* delayed gastric emptying, *LOS* length of stay, *IQR* interquartile range

Missing data: ^a^1; ^b^20; ^c^16; ^d^2

^e^Total pancreatectomies excluded

^f^Calculated among patients with pre-existing diabetes

^g^Total pancreatectomies and chronic pancreatitis excluded

Major morbidity occurred more frequently after PD (23.9%) and TP (19.8%) than after DP (8.3%) (*P* = 0.0003). The median length of stay was significantly longer for PD (10 days) and TP (11 days) than for DP (8 days) (*P* < 0.0001).

The overall postoperative mortality rate was 2.7%. After PD, the mortality rate was 4.5%, compared with 1.1% after TP and 0% after DP (*P* = 0.0079). The median age of the patients who experienced postoperative death after PD (75 years; IQR, 6 years) was significantly higher than that of the other patients in the sample (68 years; IQR, 14 years) (*P =* 0.0044). Surgical outcomes did not differ significantly, including mortality, according to final pathology (low-grade dysplasia vs high-grade dysplasia/invasive cancer).

### Predictors of Severe Surgical Complications and Classification Tree

Age, CACI score, history of acute pancreatitis, and type of surgical intervention were associated with postoperative major morbidity (CD ≥3) in the univariate analysis without adjustment for multiple testing. Only DP versus PD remained significant after application of Bonferroni’s correction and were protective against severe postoperative complications (odds ratio [OR], 0.30; 95% CI, 0.15–0.55; *P* = 0.0045).

**Fig. 1:**
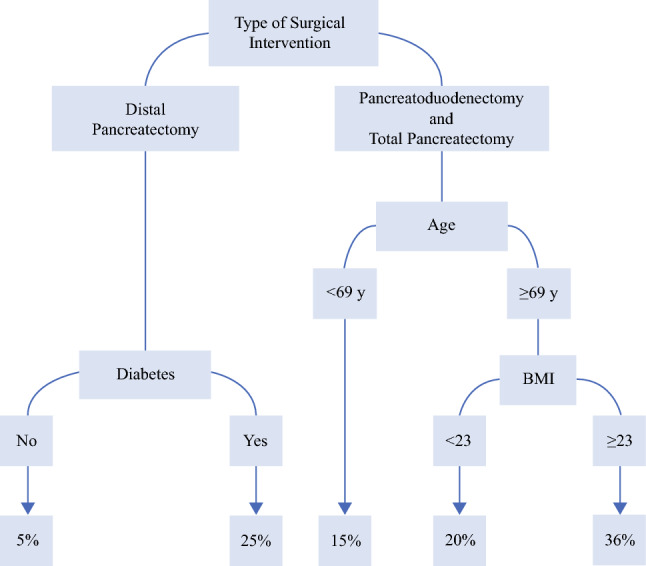
Classification tree for predicting postoperative major morbidity (Clavien–Dindo ≥3) (*n* = 524).

A classification tree model was estimated to derive a tool to predict the occurrence of major complications. The type of surgical intervention was the main variable distinguishing patients with different probabilities of severe complications (Fig. 1). Among the patients undergoing DP, the presence of preoperative diabetes appeared to define two groups with different probabilities of major morbidity (25% vs 5%). In the candidates for PD or TP, age and BMI segregated three different risk classes for major morbidity (with occurrence-of-risk probabilities of 15%, 20% and 36%). The AUC for the classification tree was 0.701 (95% CI, 0.628–0.773).

### Long-Term Outcomes

Endocrine insufficiency was evaluated in 335 patients, thus excluding those undergoing TP. New-onset diabetes occurred for 25.6% of the patients, whereas the worsening of pre-existing diabetes occurred for 66.7% of the diabetic patients. The patients with chronic pancreatitis and those undergoing TP also were excluded from the analysis of exocrine insufficiency, which was performed for 322 patients. Among these patients, 36% experienced postoperative exocrine insufficiency.

### Predictors of Endocrine Insufficiency/Diabetes and Classification Tree

Endocrine insufficiency was analyzed as a time-dependent variable. The mean time to new or worsening diabetes was 67.61 months (95% CI, 61.71–73.51 months). Overall, 64.48% (95% CI, 58.81–70.71%) of the patients were diabetes-free 5 years after surgery. The univariate analysis for the time to NOD/worsening diabetes is reported in Table S1. Age, CACI score, history of diabetes, and type of surgical intervention (DP) were associated with a higher risk of NOD/worsening diabetes in the univariate analysis, without adjustment for multiple testing. All the aforementioned factors were confirmed after application of Bonferroni’s correction, except the type of surgical resection.

A survival tree model was estimated (Fig. S1). The first variable defining two groups with different risks appeared to be the presence of preoperative diabetes. Among the patients with a history of diabetes, age and cyst size segregated three different risk classes for worsening diabetes. The C-index for the groups defined by the survival tree was 0.662 (95% CI, 0.618–0.708).

### Predictors of Exocrine Insufficiency and Classification Tree

The univariate analysis for exocrine insufficiency is reported in Table S2. The type of surgical intervention (DP) was shown to be protective against exocrine insufficiency in the univariate analysis, also after application of Bonferroni’s correction. A classification tree model was estimated (Fig. S2). The type of surgical resection was the first variable defining the two groups with different probabilities of the outcome. The patients who underwent DP presented a 36% risk of exocrine insufficiency. Among the patients who underwent PD, BMI, cyst size, and age segregated four different risk classes for exocrine insufficiency. The AUC for the classification tree was 0.759 (95% CI, 0.707–0.810).

## Discussion

The overall major postoperative morbidity of patients undergoing pancreatic resection for IPMN is similar to that for other indications. The risk of major morbidity and postoperative pancreatic insufficiency for these patients can be preoperatively stratified according to the type of intervention based on the presence of preoperative diabetes, age, BMI, and cyst size. Such preoperative risk assessment should help counseling and personalization of clinical decisions for surgical candidates, especially in the absence of preoperative features of malignancy (i.e., high-risk stigmata).

This could be the first study investigating the actual postoperative outcomes of patients undergoing surgery for IPMN, reporting disease-specific predictors of postoperative morbidity.

The original hypothesis was that surgery for IPMN may be burdened by higher rates of major postoperative morbidity, particularly POPF, because obstructive chronic pancreatitis resulting in a firm texture of the pancreas is less frequent than in other pathologies. A retrospective study based on a national database previously identified higher rates of organ space infection and sepsis after PD for benign/premalignant neoplasms than with other pathologies.^19,20^ This finding was not confirmed in this study because major morbidity after PD for IPMN did not differ from that historically encountered at this same center (23.9% vs 20.0%, respectively).^21^ Conversely, the overall morbidity after DP was higher in this series than reported in recent literature, but the rates of severe morbidity were twofold lower (8.3% vs 17.2%).^3^

Surprisingly, the overall rates of CR-POPF were lower than expected. A possible explanation of this finding may be represented by the high rate of MPD involvement encountered preoperatively. In addition to being a protective factor against the occurrence of CR-POPF after PD, a dilated MPD in IPMNs also represents a prevalent indication for surgery. Presumably for this high occurrence in this study, MPD dilation was not a significant predictor of lower major morbidity among the patients undergoing pancreatectomy for IPMNs, contrary to the reports in the literature.

Conversely, the rate of biliary fistula was strikingly high (9% after PD). Even if conservatively treated in most cases, biliary fistula can be life-threatening, especially when associated with other complications, such as POPF. The higher incidence of biliary fistula could be explained by technical difficulties associated with hepaticojejunostomy in nondilated biliary ducts (notably, only 15% of the patients were preoperatively jaundiced) because findings have demonstrated this to be the only predictive factor of its occurrence.^22^

The mortality rates after DP and TP were comparable with those reported in historical cohorts. However, the mortality rate after PD was strikingly higher than expected (4.5%). This finding should be considered with caution because it might imply a frailer population submitted to PD for IPMN. Notably, postoperative mortality after PD occurred in patients significantly older than the others. Some authors have recently suggested that surgery could be a cost-effective choice for BD-IPMN arising in the head of the pancreas.^23–25^ However, the IPMN-specific surgical outcomes and mortality reported in the current study may challenge the cost-effectiveness of PD for elderly patients who have IPMN without certain signs of malignancy.

This study aimed to identify models to predict postoperative outcomes, which could easily support physicians in the decision-making process. This kind of model has the following advantages: it easily handles eventual interaction effects between variables; it does not need to use complete data; and it generates a decision chart that is easy to use in practice. It is important to acknowledge that when decision trees are used in everyday practice, the proposed cutoffs need to be interpreted and applied with flexibility because they may be adjusted based on what is commonly used in clinical practice. However, the trees internally select the “best” cut points for the continuous variables in the predictive model, and the variables retained in the model are those with the most clinical significance. For example, despite the limited clinical applicability of the proposed cutoff, BMI was an important predictor of morbidity for patients undergoing PD or TP in the current series, increasing the risk of severe complications. Either PD or TP for IPMNs should be considered a very risky intervention for older and overweight patients, with a risk of major morbidity ranging from 20 to 36%.

Several studies have explored the impact of preoperative diabetes mellitus (DM) on the surgical outcome of pancreatectomies. Most of these studies included patients with pancreatic ductal adenocarcinoma (PDAC) and found no detrimental impact of DM on mortality or morbidity.^26–28^ We identified DM to be an important predictor of adverse outcomes after DP for IPMNs. Historically, DP is considered the less demanding procedure among pancreatic surgeries and is accompanied by fewer complications than PD or TP. This also was confirmed by the current series, in which major morbidity after DP was two- and threefold lower than after PD or TP. However, the presence of preoperative DM seems to strongly tip the balance of the surgical risk, increasing the risk of major morbidity after DP fivefold for diabetic patients. Postoperative diabetes will develop in about 25% of non-diabetic patients and worsen in most of those who already are diabetic. However, older patients with larger cysts who are already affected by preoperative diabetes comprise a group with a higher risk for worsening of this condition regardless of specific cutoffs. Conversely, although affected by multiple factors, the incidence of postoperative exocrine insufficiency remained acceptable in each risk class.

The current study had several limitations. The first limitation was the retrospective nature of the study. Second, most of the predictors selected by the model were not modifiable, except for patient BMI. Moreover, a universally accepted cutoff for overweight definition (i.e., BMI >25 kg/m^2^) did not perfectly segregate outcomes in our cohort, making its use difficult in routine clinical practice. Long-term follow-up data were not available for all the patients, possibly causing underestimation of the real burden of postoperative pancreatic insufficiency. Finally, further external validation would be required to confirm these findings.

Generally, oncologic purposes usually overtake considerations about the possible occurrence of major postoperative morbidity. This was the case of a surgically fit patient with high likelihood of harboring malignancy including invasive cancer or high-grade dysplasia, which can be predicted according to the presence of high-risk stigmata (as confirmed by the current series) and/or cytology positive for malignant or atypical cells. However, in nearly one third of the patients undergoing surgery for IPMNs (including the current series), low-grade dysplasia is found at final pathology, and this chance is increased when worrisome features alone are present preoperatively.^29^ Notably, complications and mortality after surgical resection did not spare patients who did not harbor malignancy. For this reason, the prediction of surgical outcomes remains of paramount importance for both patient selection and counseling. Whenever the preoperative features of the cyst are not certainly suggestive for the presence of malignancy and the patient is diabetic (in case of DP) or older and overweight (in case of PD/TP), surveillance may be considered a better option due to increased mortality. Finally, an elevated incidence of worsening diabetes must be considered for older patients with larger cysts who already are affected by preoperative diabetes.

In conclusion, the current study depicted the actual surgical outcomes of resected IPMNs in referral centers. Preoperative variables with a potential impact on postoperative morbidity were integrated into a model that can be used in clinical practice. Because mortality remains a major issue once older individuals undergo surgery for IPMN, a preoperative disease-specific stratification of surgical risk is clinically meaningful in this scenario, allowing clinicians to personalize the surgical indication and improve counseling for patients with uncertain risk of malignancy.

## Supplementary Information

Below is the link to the electronic supplementary material.

Supplementary file1 (TIF 4383 kb)

Supplementary file2 (TIF 2835 kb)

Supplementary file3 (DOCX 23 kb)
