# Supplementary figures and images for: Surgery for Intraductal Papillary Mucinous Neoplasms of the Pancreas: Preoperative Factors Tipping the Scale of Decision-Making
Source: Ann Surg Oncol. 2022 Jan 24;29(5):3206–14. doi: 10.1245/s10434-022-11326-5 (PMC8989932; doi:10.1245/s10434-022-11326-5)

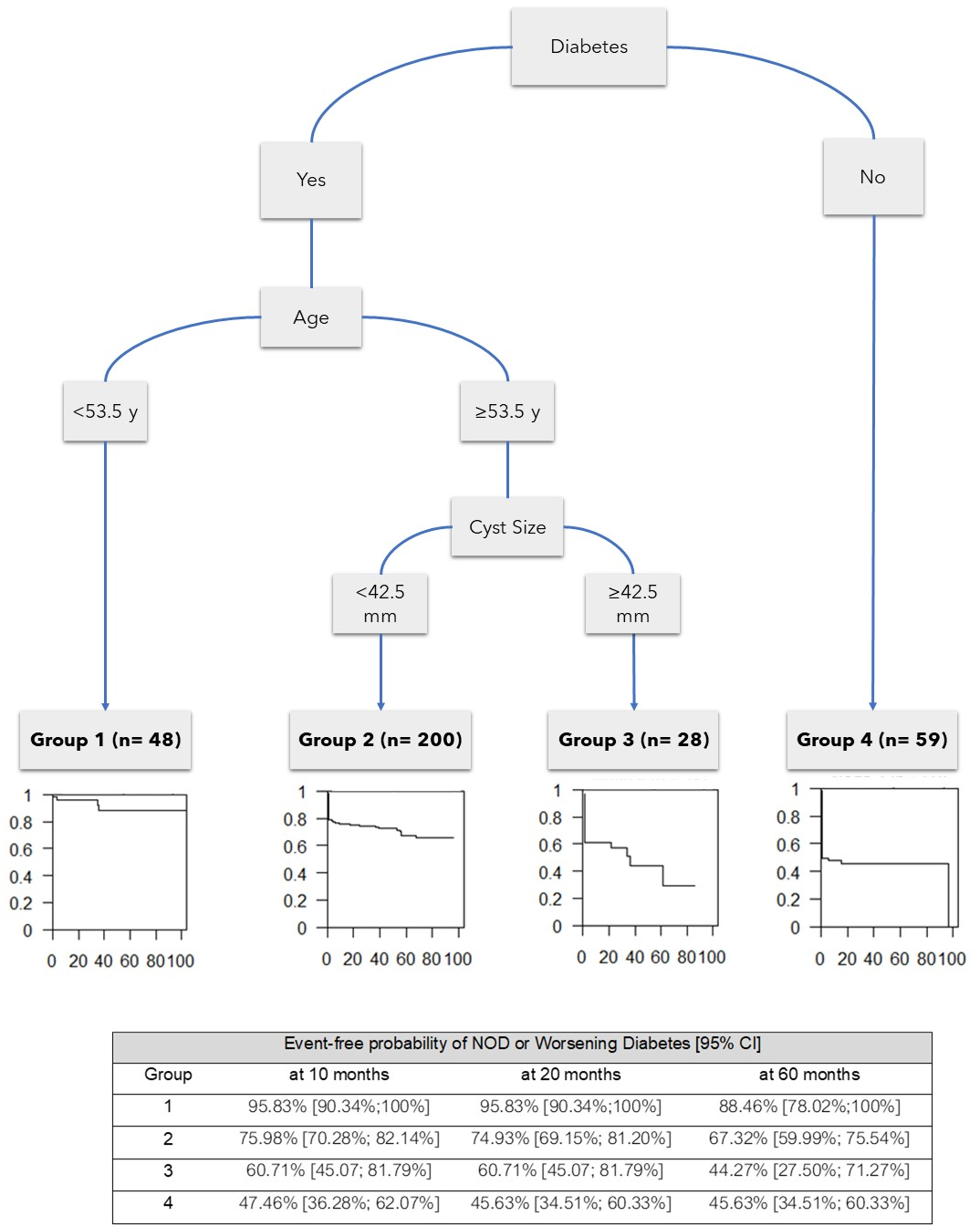

Supplement: Supplementary file 1 — Supplementary file1 (TIF 4383 kb) [file 10434_2022_11326_MOESM1_ESM.tif]

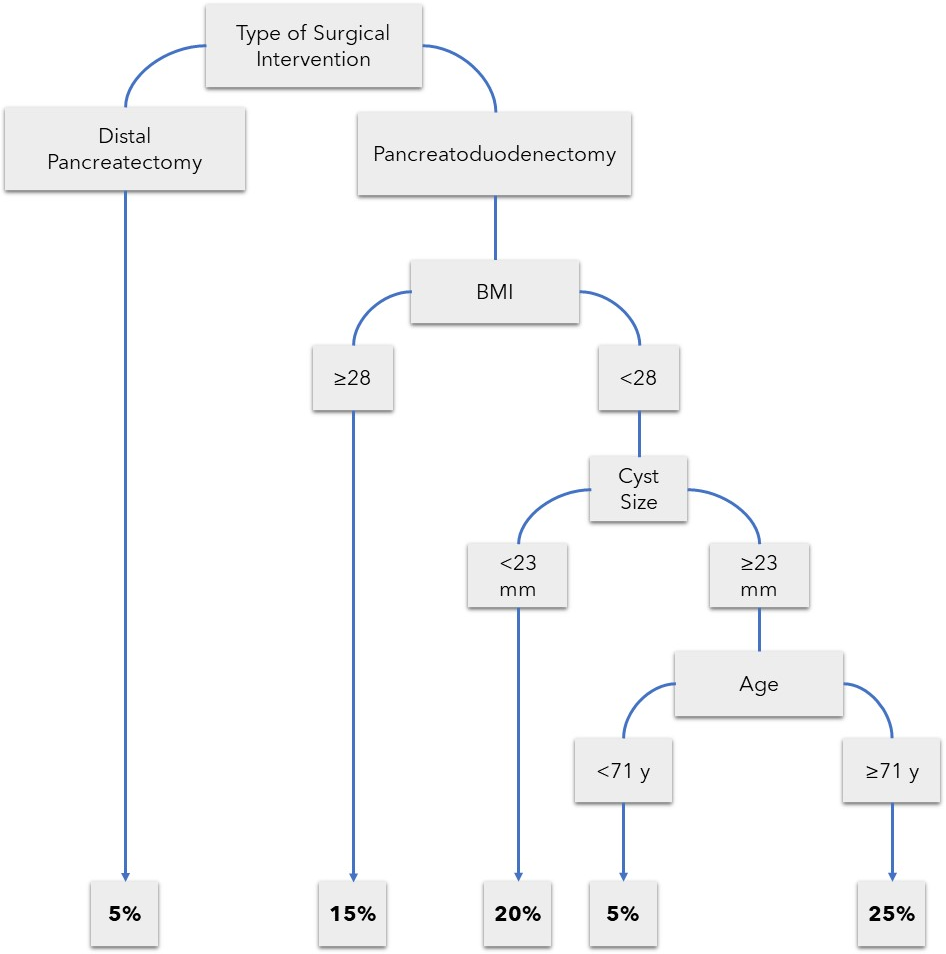

Supplement: Supplementary file 2 — Supplementary file2 (TIF 2835 kb) [file 10434_2022_11326_MOESM2_ESM.tif]
