# Supplementary material for: Surgery for Intraductal Papillary Mucinous Neoplasms of the Pancreas: Preoperative Factors Tipping the Scale of Decision-Making
Source: Ann Surg Oncol. 2022 Jan 24;29(5):3206–14. doi: 10.1245/s10434-022-11326-5 (PMC8989932; doi:10.1245/s10434-022-11326-5)
Supplement: Supplementary file 3 — Supplementary file3 (DOCX 23 kb) [file 10434_2022_11326_MOESM3_ESM.docx]

|  | **Supplementary Table 1** – Univariate Cox’s proportional hazard regression analysis for predictors of time to NOD or Worsening Diabetes (N= 335) | | | | | | |
| --- | --- | --- | --- | --- | --- | --- | --- |
|  | |  | N | HR | 95% CI HR | p-value | adj. p-value |
| Age | |  | 335 | 1.03 | 1.01-1.05 | **0.0022** | **0.0415** |
| BMI | |  | 249 | 1.01 | 0.95-1.07 | 0.8475 | 1.0000 |
| Current Smoker | |  | 333 | 0.73 | 0.41-1.27 | 0.2611 | 1.0000 |
| Comorbidities | |  |  |  |  |  |  |
| Diabetes | |  | 333 | 2.68 | 1.77-4.05 | **<0.0001** | **0.0001** |
| Hypertension | |  | 335 | 1.36 | 0.93-1.99 | 0.1110 | 1.0000 |
| Solid tumor | |  | 335 | 0.95 | 0.53-1.69 | 0.8527 | 1.0000 |
| CACI | |  | 335 | 1.21 | 1.11-1.32 | **<0.0001** | **0.0002** |
| ASA ≥3 *vs* <3 | |  | 297 | 1.17 | 0.75-1.84 | 0.4867 | 1.0000 |
| Symptoms | |  |  |  |  |  |  |
| Abdominal pain | |  | 335 | 0.95 | 0.62-1.44 | 0.7920 | 1.0000 |
| Weight loss | |  | 335 | 1.22 | 0.77-1.92 | 0.3971 | 1.0000 |
| Acute pancreatitis | |  | 335 | 0.72 | 0.44-1.15 | 0.1685 | 1.0000 |
| Cyst size | |  | 265 | 1.01 | 1.00-1.02 | 0.1740 | 1.0000 |
| MPD dilatation ≥5mm | |  | 289 | 1.26 | 0.73-2.16 | 0.4037 | 1.0000 |
| Solid component | |  | 333 | 1.18 | 0.80-1 .75 | 0.4006 | 1.0000 |
| Chronic pancreatitis | |  | 333 | 1.22 | 0.54-2.78 | 0.6354 | 1.0000 |
| Type of surgery: DP vs PD | |  | 335 | 1.63 | 1.12-2.37 | **0.0114** | 0.2161 |
| Final pathology | |  | 335 |  |  |  |  |
| MT *vs* MD-IPMN | |  |  | 1.02 | 0.62-1.70 | 0.9276 | 1.0000 |
| BD *vs* MD-IPMN | |  |  | 1.30 | 0.69-2.46 | 0.4153 | 1.0000 |
| Abbreviations: BMI; body mass index; CACI, Charlson-age comorbidity index; ASA, American society of anesthesiologists; MPD, main pancreatic duct; DP, distal pancreatectomy; PD, pancreaticoduodenectomy; MT, mixed-type; MD, main-duct; BD, branch-duct. | | | | | | | |

|  | **Supplementary Table 2** – Univariate logistic regression analysis for predicting exocrine insufficiency after pancreatic resection (N=322) | | | | | | |
| --- | --- | --- | --- | --- | --- | --- | --- |
|  | | N |  | OR | 95% CI OR | p-value | adj. p-value |
| Age | | 322 |  | 1.01 | 0.99-1.04 | 0.2470 | 1.0000 |
| BMI | | 237 |  | 0.92 | 0.85-0.99 | 0.0247 | 0.4202 |
| Current Smoker | | 320 |  | 2.08 | 1.07-4.32 | 0.0379 | 0.6444 |
| Comorbidities | |  |  |  |  |  |  |
| Diabetes | | 320 |  | 1.47 | 0.8-2.78 | 0.2254 | 1.0000 |
| Hypertension | | 322 |  | 0.82 | 0.52-1.28 | 0.3792 | 1.0000 |
| Solid tumor | | 322 |  | 0.75 | 0.39-1.5 | 0.4126 | 1.0000 |
|  | |  |  |  |  |  |  |
| CACI | | 322 |  | 1.02 | 0.91-1.15 | 0.7857 | 1.0000 |
| ASA ≥3 *vs* <3 | | 288 |  | 1.05 | 0.61-1.84 | 0.8627 | 1.0000 |
| Symptoms | |  |  |  |  |  |  |
| Abdominal pain | | 322 |  | 1.09 | 0.66-1.83 | 0.7460 | 1.0000 |
| Weight loss | | 322 |  | 1.57 | 0.87-2.93 | 0.1401 | 1.0000 |
| Acute pancreatitis | | 322 |  | 0.9 | 0.52-1.57 | 0.7055 | 1.0000 |
| Cyst size | | 256 |  | 1.01 | 1.00-1.03 | 0.1194 | 1.0000 |
| MPD dilatation ≥5mm | | 280 |  | 0.92 | 0.48-1.70 | 0.7867 | 1.0000 |
| Solid component | | 320 |  | 0.94 | 0.58-1.52 | 0.7947 | 1.0000 |
| Type of surgery: DP vs PD | | 322 |  | 0.16 | 0.09-0.26 | **<0.0001** | **<0.0001** |
| Final pathology | | 322 |  |  |  |  |  |
| MT *vs* MD-IPMN | |  |  | 0.96 | 0.51-1.74 | 0.8902 | 1.0000 |
| BD *vs* MD-IPMN | |  |  | 0.54 | 0.24-1.18 | 0.1244 | 1.0000 |
| Abbreviations: BMI; body mass index; CACI, Charlson-age comorbidity index; ASA, American society of anesthesiologists; MPD, main pancreatic duct; DP, distal pancreatectomy; PD, pancreaticoduodenectomy; MT, mixed-type; MD, main-duct; BD, branch-duct. | | | | | | | |
